# Effects of continuous and rotational cropping practices on soil fungal communities in pineapple cultivation

**DOI:** 10.7717/peerj.13937

**Published:** 2022-09-06

**Authors:** Jing Chen, Hui Zeng

**Affiliations:** 1 South Subtropical Crops Research Institute, Chinese Academy of Tropical Agricultural Sciences, Zhanjiang, Guangdong, China; 2 Key Laboratory of Tropical Fruit Tree Biology, Ministry of Agriculture, Zhanjiang, Guangdong, China

**Keywords:** Fungal, Pineapple, Diversity analysis, Soil

## Abstract

**Background:**

Rotational cropping practices can change the fungal structure and diversity of cropping soil, and these changes can promote crop development. However, only a few studies have explored the effects of rotational cropping of pineapple on soil fungal diversity.

**Methods:**

In this study, we investigated fungal diversity in continuous and rotational cropping soil of pineapple in Xuwen and Leizhou of China in summer and winter through high throughput sequencing of the fungal internal transcribed spacer region.

**Results:**

The diversity and richness of the fungal community were observed to be significantly increased after rotational cropping in Xuwen and Leizhou in summer, whereas no changes were observed in winter. Furthermore, Ascomycota, Basidiomycota, Zygomcota, and Chytridiomycota were the dominant phyla, and *Chaetomium, Penicillium, Fusarium, Trichoderma*, and *Cryptococcus* were the dominant genera in the continuous and rotational cropping soil of pineapple, respectively, in both summer and winter. *Chytridiomycota* at phylum level and *Gibberella* at genus level were observed in rotational cropping soil; however, *Ascomycota* at the phylum level and *Chaetomium* at the genus level were the most abundant fungi, and their abundance dramatically decreased in continuous cropping soil. Redundancy analysis revealed that rotational cropping reduced the correlation between environmental parameters and the fungal community in winter. In addition, several fungal biomarkers were found in Xuwen in both continuous and rotational cropping soil samples, including *Sporobolomyces, Aspergillus*, Corynascus sp JHG 2007, and *Corynascus* at the genus level, *Penicillium* and fungal sp p1s11 at the species level in rotational cropping soil, and ales family Incertae sedis and *Sordariomycetes* at the class level in continuous cropping soil. These results revealed the changes in the structure and diversity of fungal community in continuous and rotational cropping practices for pineapple cultivation, which may be associated with crop yield and quality.

## Introduction

Continuous cropping causes an increase in the number of soil pathogens and a continuous deterioration of the microbial ecological environment (Zhu et al., 2020; Lyu et al., 2020; Wang et al., 2020). It could also lead to an imbalance of land nutrients and a decline in sustainable use capacity, which further affects crop yield and quality. Pineapple, *Ananas comosus* (L.) Merr. belongs to the *Bromeliaceae* family, and it is the third most important tropical fruit found in almost all tropical and subtropical regions of the world (Moyle et al., 2005; Zhu et al., 2018). Problems caused by the long-term continuous cropping of pineapple inhibit the growth of pineapples and may induce the occurrence of various diseases, such as pineapple heart rot (Lechaudel et al., 2018; Shen et al., 2013). This has become a major problem that restricts the development of the pineapple industry in most countries. The geographical scope of pineapple cultivation is inherently limited and the pineapple industry may face a decline if effective measures are not promptly taken. Therefore, studying the effective prevention and control mechanisms for problems associated with continuous cropping of pineapple is of great significance for the sustainable development of the pineapple industry.

Various farming modes have been used to overcome the biological challenges of continuous cropping. Among them, rotational cropping is an effective and important prevention and control method that can change the microbial diversity as well as the physical and chemical properties of continuous cropping soil, including organic matter, nutrients, and other physical and chemical characteristics (Yang et al., 2019; Li et al., 2019). Xuwen and Leizhou, located in the Leizhou Peninsula, Guangdong, are the main areas for pineapple planting in China, accounting for 40% and 50% of China’s pineapple planting area and output, respectively (Lu et al., 2014; He et al., 2015). Pineapple tends to grow better with higher growth and yield in rotational cropping soil than in continuous cropping soil. In our previous study, we found that leaf nitrogen, chlorophyll content, and yield of pineapple under rotational cropping were significantly higher than those under continuous cropping (Chen, Gong & Xu, 2020). It has been demonstrated that crop rotation practices effectively alleviate the biological challenges of continuous cropping by maintaining the diversity and activity of soil microorganisms, inhibiting harmful microorganisms that are easy to multiply under continuous cropping mode, and increasing crop yields (Wang et al., 2016; Du et al., 2020; He et al., 2019a). Several studies have found that rotational planting patterns change the structure and abundance of soil microbial communities and increase crop yields (He et al., 2019b; Paungfoo-Lonhienne et al., 2017; Wahbi et al., 2016). It has also been demonstrated that continuous and rotational cropping practices affect soil bacterial communities in pineapple cultivation (Chen, Gong & Xu, 2020). However, few studies have focused on the effect of rotational planting of pineapple on fungal diversity. Therefore, in the present study, analysis of fungal diversity in continuous and rotational cropping practices for pineapple cultivation was performed using amplicon-based high-throughput sequencing. The aim of this study was to unveil the fungal communities associated with different cropping strategies in pineapple cultivation.

## Materials and Methods

### Collection of soil samples and measurement of the physicochemical properties

Samples were obtained from inter-row soil (*i.e*., soil between planting rows) of pineapple (variety: Comte de Paris) crops in Leizhou and Xuwen (Guangdong, China), according to our previous study (Chen, Gong & Xu, 2020). For continuous cropping, the pineapple was planted in succession for at least 18 years. For rotational cropping in Leizhou, three of the five farmlands used pineapple–banana rotation cropping, while the other two used pineapple–capsicum cropping. The time scale of pineapple–banana crop rotation was 16 months for pineapple and 12 months for banana, while that of pineapple–capsicum was 16 months for pineapple and 5 months for capsicum. For rotational cropping in Xuwen, all the five pineapple rotational farmlands involved pineapple–sugarcane cropping. The time scale of pineapple–sugarcane crop rotation was 16 months for pineapple and 12 months for sugarcane. Between each rotation, the stubble of previous plants was destroyed by tractor, and was retted in soil for 3 months. Subsequently, the soil was ploughed by tractor and retted before raking. The next crop was then planted at the farmlands. During cropping, the same fertilization/soil management strategy was applied to both continuous and rotational cropping. Briefly, the foliation fertilizer was firstly applied at about 50 days after the planting of pineapple seedlings, and then applied every other month. Further, the chemical fertilizer (375 kg urea, 300 kg potassium chloride, and 300 kg compound fertilizer per hectare) was applied between rows in every spring. Notably, the selection of rotational cropping systems was in accordance with the prevalence of different rotational systems in these two locations. In total, 80 soil samples were collected in a random zig-zag manner at 0–15 cm depth, categorized into eight groups (continuous cropping soils of Leizhou in summer (LCS) and winter (LCW), rotational cropping soils of Leizhou in summer (LRS) and winter (LRW), continuous cropping soils of Xuwen in summer (XCS) and winter (XCW), and rotational cropping soils of Xuwen in summer (XRS) and winter (XRW)). Each group contained 10 samples from five farmlands (two samples in each farmland) and each sample were a mixed of ten subsamples (Table S1). The physicochemical parameters (pH, total organic matter (TOC), total nitrogen (TN), available nitrogen (N), phosphorus (P), and potassium (K)) of the soil were measured as previously reported (Chen, Gong & Xu, 2020).

### DNA extraction and polymerase chain reaction (PCR)

We extracted of DNA from 0.5 g samples of soil according to the manufacturer’s instructions of Power Soil DNA kit (Qiagen, Hilden, Germany). And then we purified the combined duplicate DNA extractions using a DNA gel extraction kit (Axygen, Union City, CA, USA). A BioTek Epoch Microplate Spectrophotometer (Winooski, VT, USA) was used to check the DNA concentration of each sample (Chen, Gong & Xu, 2020).

The extracted DNA samples were used as templates for PCR amplification targeting the internal transcribed spacer 1(ITS1) region of the fungus. The ITS region was targeted using the primers ITS1F (5-CTTGGTCATTTAGAGGAAGTAA-3) and ITS2 (5-GCTGCGTTCTTCATCGATGC-3) (Orgiazzi et al., 2012). The reaction volume for PCR analysis was 25 μL, comprising 10 μL 2×Taq Master Mix, 1 μL template DNA, 0.5 μL forward/reverse primer, respectively. The amplification procedure was as follows: 95 °C for 5 min; 95 °C for 30 s, 58 °C, 30 s and 72 °C, 30 s for 42 cycles. PCR products were stored at −20 °C immediately after the end of the reaction. Using the Illumina HiSeq 2500 platform (Illumina, San Diego, CA, USA) at Biomarker Technologies Co, Ltd. (Beijing, China), the PCR products were used to construct the sequencing libraries according to the standard protocol.

### Raw data processing

The sequencing reads of all samples were rarefied to read number of the sample with lowest sequencing reads by a custom script. Then the rarefied paired-end data was merged into a sequence of tags based on the overlap relationship, and the quality of the reads and the merged effect were quality-controlled and filtered. FLASH v1.2.7 software was used to stitch the reads of each sample through overlap and obtain the original tags (Magoč & Salzberg, 2011), and Trimmomatic v0.33 software was used to filter the spliced raw tags to obtain clean tags (Bolger, Marc & Bjoern, 2014). Finally, effective tags were obtained by removing the chimera sequence using UCHIME v4.2 software (Edgar et al., 2011).

### Taxonomic analysis

Operational taxonomic units (OTUs) were clustered using UCLUST in QIIME (version 1.8.0) software at a similarity level of 97% (Caporaso et al., 2010). Taxonomic annotation of OTUs was performed based on the UNITE (fungal) taxonomic database (version 8.0) (Nilsson et al., 2018). The Venn graph shows the number of common and unique OTUs between samples and visually shows the overlap of OTUs between samples. Combined with the species represented by OTUs, it is possible to find common microorganisms in different environments. The Venn diagrams for each classification level were drawn using the R software VennDiagram (Chen & Boutros, 2011).

### Fungal diversity analysis

Alpha diversity reflects the richness and diversity of a single sample with four measurement indicators, including the Shannon index, chao 1 index, observed species index, and PD whole tree index. The Chao1 index was used to evaluate species abundance, while the Shannon index was used to evaluate species diversity and was influenced by the abundance of species in the sample community and community evenness. The alpha diversity index of the samples was evaluated using Mothur (version v. 1.30) software (Schloss et al., 2009) and compared among different groups with one-way ANOVA followed by Duncan’s multiple range test. Beta diversity analysis based on OTU relative abundance was carried out to examine the differences in microbial community structure among different samples by calculating the ecological distance of Bray Curtis, Unifrac, etc, and pairwise PERMANOVA was performed to estimate the statistical significance between different groups. The QIIME2 software package (https://docs.qiime2.org/2022.8/) was used to calculate the beta diversity distance, and the module “cmdscale” in R software was used to perform principal coordinate analysis (PCoA).

### Statistical analysis

The difference in relative abundance of fungi among different groups was calculated with one-way ANOVA followed by Duncan’s multiple range test. The correlation between the fungal community and environmental parameters in winter was analyzed by redundancy analysis (RDA) using Canoco 5.0 (Ter Braak & Smilauer, 2012). Significant differences between groups were mainly used to find biomarkers among different groups by performing linear discriminant analysis (LDA) effect size (LEfse) based on the normalized abundance table of each species level (Segata et al., 2011). The biomarker screening criteria was LDA score >4.

## Results

### Overview of the sequencing data

**Figure 1:**
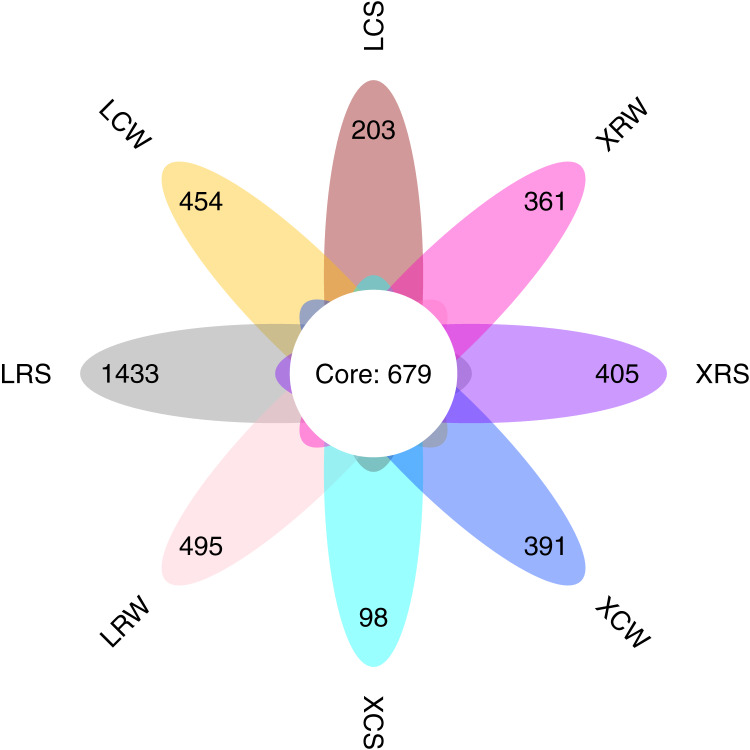
Number of common and unique operational taxonomic unit (OTU) presented by Venn diagram.

We performed ITS sequencing to investigate the fungal communities in the continuous and rotational cropping soil of pineapple cultivation. The sequencing produced an average of 67,932 (ranging from 49,838 to 72,523) effective tags for each sample (Table S2). All the effective tags were clustered into 12,346 OTUs assigned to the taxonomy of the fungi. The average OTU number for all detected samples was 1,329 (ranging from 677 to 2,913). The number of common fungal OTUs shared between the continuous and rotational cropping soil of pineapple in summer and winter was 679 (Fig. 1). The unique OTUs in LRS, LCS, XRS, XCS, LRW, LCW, XRW, and XCW were 1,433, 203, 405, 98, 495, 454, 361, and 391, respectively. Consistent with the results of fungal community composition in the continuous and rotational cropping soil of pineapple, these results demonstrated that the number of OTUs was significantly increased in the rotational cropping soil of pineapple in summer. Taken together, these results demonstrate that rotational cropping caused structural and diverse changes in the planting soil of pineapple.

### Diversity analysis of fungi in the continuous and rotational cropping soil of pineapple

**Figure 2:**
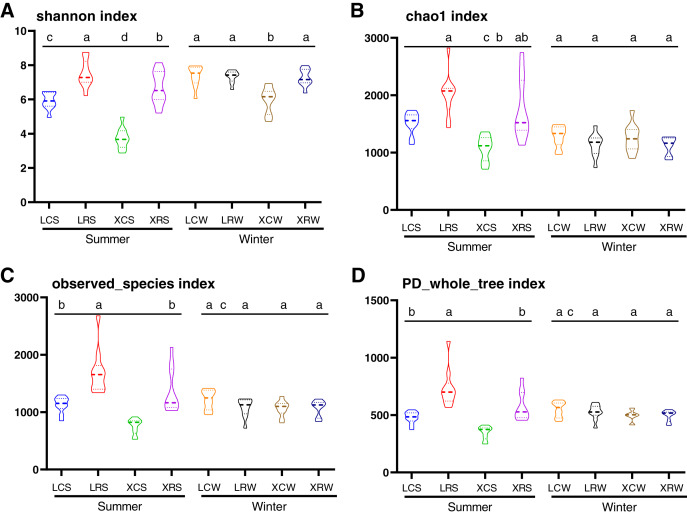
Alpha diversity index analysis in the samples from continuous and rotational cropping practices soil, including Shannon index (A), Chao1 index (B), observed species index (C) and PD whole tree index (D). XCS, samples from continuous cropping soil at Xuwen in summer; XCW, samples from continuous cropping soil at Xuwen in winter; XRS, samples from rotation cropping soil at Xuwen in summer; XRW, samples from rotation cropping soil at Xuwen in winter; LCS, samples from continuous cropping soil at Leizhou in summer; LCW, samples from continuous cropping soil at Leizhou in winter; LRS, samples from rotation cropping soil at Leizhou in summer; LRW, samples from rotation cropping soil at Leizhou in winter. Values sharing the same letter are not significantly different at *P* < 0.05 (one-way ANOVA and Duncan’s multiple range test).

In the present study, the Shannon index, Chao1 index, observed species index, and PD whole tree index were used to evaluate species diversity and richness (Table S3). These indices indicated that the richness and diversity of fungi was significantly higher in the rotational cropping soil of pineapple than in the continuous cropping soil (Duncan’s multiple range test, *P* < 0.05). However, there was no significant difference between pineapple–banana (LRS01, LRS02, LRS03, LRS06, LRS07, and LRS08) and pineapple–capsicum (LRS04, LRS05, LRS09, and LRS10) rotation cropping farms at Leizhou (Table S3). In addition, rotational cropping and continuous cropping soil in winter showed no significant difference (Figs. 2A–2D). These results demonstrated that both species richness and species diversity were mostly affected by the cropping modes (continuous or rotational cropping).

**Figure 3:**
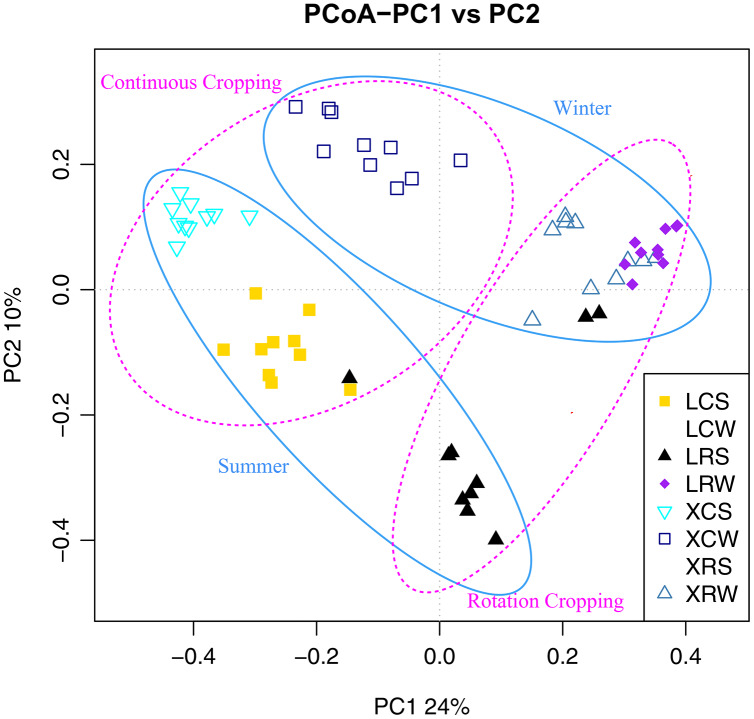
Principal coordinates analysis (PCoA) plot depicted the Bray–Curtis distance of fungal communities in the soil samples from continuous and rotational cropping practices in summer and winter.

To confirm the differences in the fungal communities between continuous cropping and rotational cropping in winter or summer, principal coordinate analysis (PCoA) based on the Bray–Curtis dissimilarity index was carried out. The results showed that the two main coordinates extracted explained 34% of the variation. Further analysis showed that the distribution of the samples was also significantly different between summer and winter and between continuous and rotational cropping (PERMANOVA, R^2^ = 0.46, *P* ≤ 0.001; Fig. 3, Table S4), only except for the distance between continuous cropping and rotational cropping samples of Leizhou in the winter. These results demonstrate that a significant difference exists between continuous cropping and rotational cropping.

### Community composition of fungi in the continuous and rotational cropping soil of pineapple

**Figure 4:**
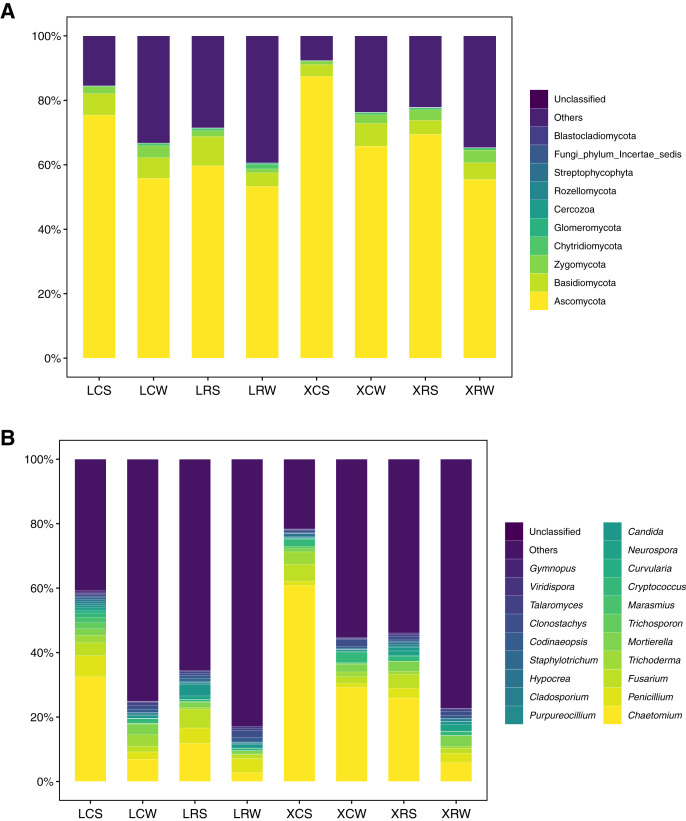
Taxonomic summary of the soil fungal community under continuous and rotational cropping practices at the phylum level (A) and genus level (B).

The structure and diversity analysis of fungal community showed that fungal diversity significantly increased in continuous cropping soil. At the phylum level, a total of 11 groups were detected at >1% abundance (Table S4). *Ascomycota* (43.34–90.93%), *Basidiomycota* (1.98–22.97%), *Zygomcota* (0.44–6.96%), and *Chytridiomycota* (0.19–5.17%) were the dominant phyla in the continuous and rotational cropping soil of pineapple both in the summer and winter. Among them, *Ascomycota* was the most abundant, accounting for more than 50%, which dramatically increased in the continuous cropping soil in summer (one-way ANOVA, *P* < 0.01). However, in the winter, no obvious changes were observed for *Ascomycota* in the continuous and rotational cropping soil of pineapple (Fig. 4A). In addition, 26 groups were detected at >1% abundance for fungi at the genus level (Table S5), and the dominant genera of the continuous and rotational cropping soil of pineapple both in the summer and winter were *Chaetomium*, *Penicillium, Fusarium, Trichoderma*, and *Cryptococcus* (Fig. 4B). Further analysis showed that *Chaetomium* richness was significantly increased in continuous cropping soil in both the summer and winter (one-way ANOVA, *P* < 0.01). However, it was dramatically lower in the winter than in the summer (Fig. 4B). In addition, our analysis showed that *Chytridiomycota* at the phylum level and *Gibberella* at the genus level increased after rotational cropping both in the winter and summer (Fig. 4).

### Relationship between physicochemical parameters and fungal community

**Figure 5:**
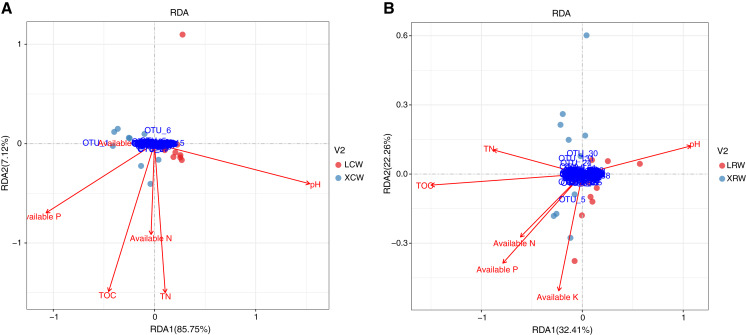
Redundancy analysis (RDA) of the relationship between fungal community structure and physicochemical parameters in the continuous (A) and rotational (B) cropping soils of pineapple in winter. Red dots represent LCW group samples and cyan dots represent XCW group samples. Blue dots represent the significant operational taxonomic units in RDA analysis. Red arrows represent physicochemical parameters of soils. LCW, samples from continuous cropping soil at Leizhou in winter; XCW, samples from continuous cropping soil at Xuwen in winter.

The correlation between the structure of the fungal community and physicochemical parameters in the continuous and rotational cropping soils of pineapple in winter was investigated using redundancy analysis. In continuous cropping soil, RDA1 and RDA2 accounted for 85.75% and 7.12% of the changes in the structure of fungal community, respectively (Fig. 5A, Table S6), indicating that the detected physicochemical parameters accounted for most of the structural changes in the fungal community. Among the significantly correlated parameters, pH showed the greatest influence on fungal community in continuous cropping soil, followed by total organic matter (TOC), total nitrogen (TN), and available phosphorus (P). Available nitrogen (N) and potassium (K) had no significant correlation with fungal community in continuous cropping soil. In rotational cropping soil, RDA1 and RDA2 contributed to 32.41% and 22.26% of the variations in the structure of fungal community, respectively (Fig. 5B, Table S7). Moreover, TOC and pH significantly influenced the fungal community, while the other parameters did not significantly correlate with the fungal community in rotational cropping soil.

### Fungal biomarkers in the continuous and rotational cropping soil of pineapple

**Figure 6:**
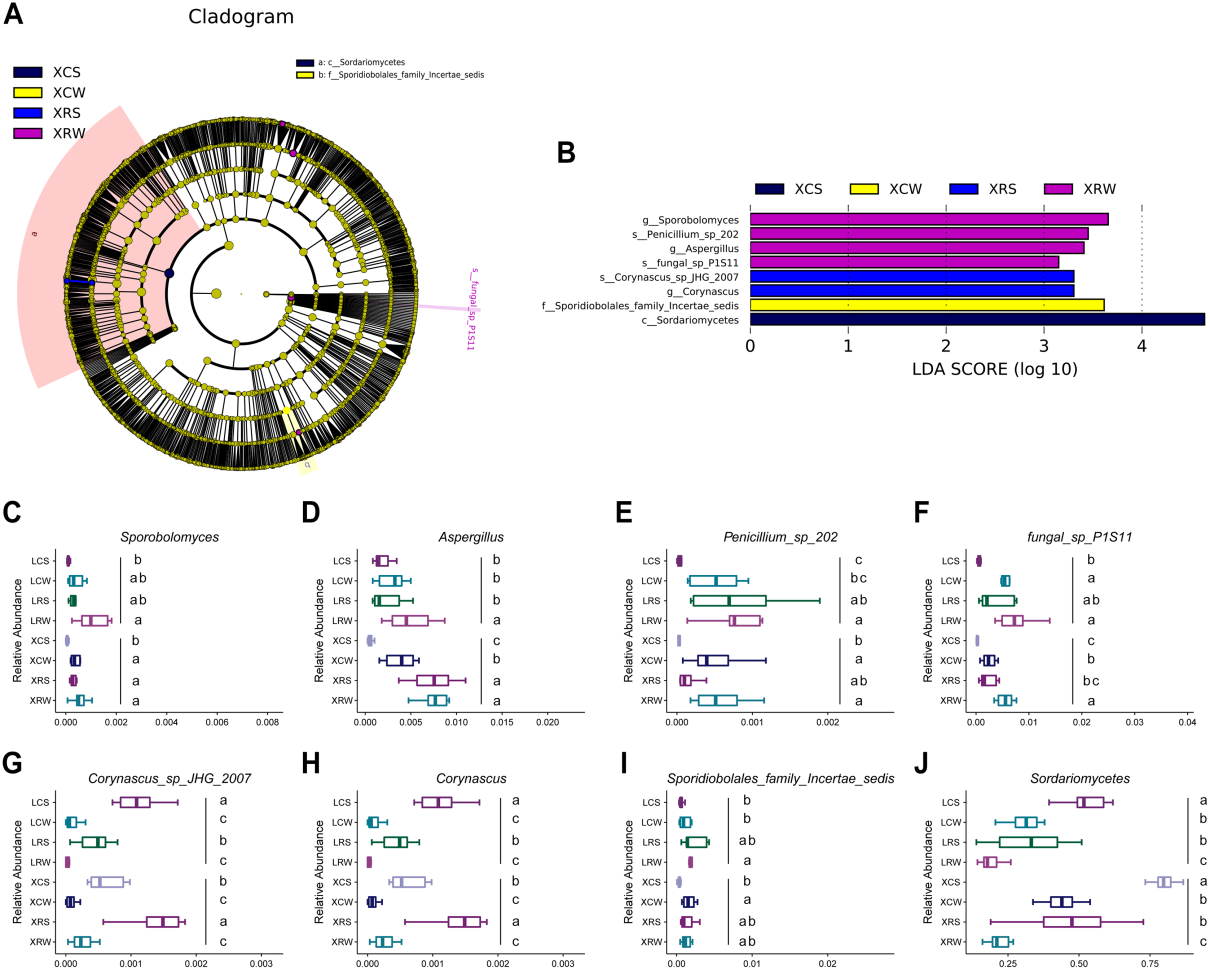
Linear discriminant analysis (LDA) effect size of the species difference in continuous and rotational cropping practices in pineapple cultivation. (A) Cladogram indicating the phylogenetic distribution of the soil fungal community associated with rotation cropping and continuous cropping in summer and winter; the phylum, class, order, family, and genus levels are listed in order from inside to outside of the cladogram and the labels for levels of family and genus are abbreviated by a single letter. (B) Bar chart of the linear discriminant analysis (LDA) scores between rotation and continuous cropping practices in summer and winter, *p* < 0.05; LDA score 3.0. (C–J) Eight fungal biomarkers among four groups. Values sharing the same letter are not significantly different at *P* < 0.05 (one-way ANOVA and Duncan’s multiple range test).

We have demonstrated the effects of rotational cropping practices on the structure and diversity of soil fungal community in summer and winter in Xuwen and Leizhou. LEfSe algorithms were used to explore the fungal biomarkers in the planting soil of pineapple. The analysis showed that eight fungal groups had significant differential distribution among the XCS, XCW, XRS, and XRW groups, including *Sporobolomyces* and *Aspergillus* at the genus level and *Penicillium* and *fungal sp p1s11* at the species level in rotational cropping soil at Xuwen in winter; *Corynascus sp. JHG 2007* at the species level and *Corynascus* at the genus level in rotational cropping soil at Xuwen in the summer; *Sporidiobolales family Incertae sedis* in continuous cropping soil at Xuwen in the winter; and *Sordariomycetes* at the class level in continuous cropping soil at Xuwen in the summer (Figs. 6A and 6B). However, no significantly differentially distributed fungal groups were found in Leizhou groups. The distribution of the eight fungal groups in the two pineapple cropping practices in summer and winter are shown in Figs. 6C–6J. Notably, the biomarkers of XRW (*Sporobolomyces*, *Aspergillus*, *Penicillium*, and *fungal sp. p1s11*) and XCS (*Sordariomycetes*) groups also showed similar trend in LRW and LCS groups (one-way ANOVA and Duncan’s multiple range test, *P* < 0.05) (Figs. 6C–6F and 6J), whereas the biomarkers of XRS group were more abundant in LCS group rather than in LRS group (one-way ANOVA and Duncan’s multiple range test, *P* < 0.05) (Figs. 6G and 6H).

## Discussion

Soil fungi are important regulators of material circulation, organic matter decomposition, and plant mineral nutrients in the soil ecosystem (Lecomte et al., 2016; Burke et al., 2009). They play an important role in the prevention and control of pests and diseases. However, some of these fungi appear in the form of pathogenic bacteria and invade crops resulting in crop diseases (Lecomte et al., 2016; van der Heijden, Bardgett & van Straalen, 2008). Previous studies have shown that the soil fungal community is affected by many factors, not only by the living environment and substrate, but also by crop type, soil type, and measures for farmland management (Egbe, Oyetibo & Ilori, 2020; Yang et al., 2020). Continuous and rotational cropping methods are important farmland management systems that could affect the community structure of soil fungi in the farmland of crops, including pineapple. However, the structure and diversity of fungi in continuous and rotational cropping soil of cultivated pineapple have not been explored. Therefore, this study investigated the effects of rotational and continuous cropping on the structure of soil fungal community and the influence on some functional flora in summer and winter at Xuwen and Leizhou. This will explain the internal connection between different crop strategies and changes in the structure of soil fungal community.

Under the conditions of rotational and continuous cropping, different crops produce root exudates that inevitably induce different rhizosphere effects and in turn cause changes in the fungal community structure (Lauber et al., 2008). Different studies have shown that the root system of peanuts secretes phenolic acids that reduce the relative abundance of *Mortierella* sp. and *Geminibasidium hirsutum*, while increasing the relative abundance of *Fusarium oxysporum*, *Bionectria ochroleuca*, and *Phoma macrostom* (Hua et al., 2014). It has also been demonstrated that pineapple and sugarcane rotation not only changes the amount of soil microorganisms but also increases the species diversity (Jingen, 2010; Zheng et al., 2003). However, the specific microbial species in pineapple soil affected by rotational cropping remain unclear. The overall activity of soil microorganisms and biological activity are enhanced with rotational cropping, leading to an increase in soil available nutrients. In the present study, alpha diversity and taxonomic summary of soil fungal community under continuous and rotation cropping practices showed that both species richness and species diversity were significantly elevated in the soil of crop rotation practices, compared with those in the soil of continuous cropping practices (Fig. 2). Our results also showed that *Chytridiomycota* at the phylum level and *Gibberella* at the genus level appeared in rotational cropping soil both in the winter and summer (Fig. 4, Tables S4 and S5). However, *Ascomycota* at the phylum level and *Chaetomium* at the genus level were the most abundant fungal species in continuous cropping soil in summer. Li et al. (2016) demonstrated that the relative abundance of *Ascomycota* and *Zygomycota* increased in pepper soil in long-term continuous cropping (Li et al., 2016). *Ascomycota* can cause *Fusarium* wilt and *Verticillium* wilt in several crops or vegetables, such as cotton, potatoes, wheat, and chili (Davis et al., 2006); whereas *Gibberella* can promote plant growth (Lian et al., 2018; Song et al., 2018). These results demonstrated that the decrease in *Ascomycota* could reduce the harm caused by *Ascomycota*, and *Gibberella* promoted the growth of pineapple in rotational cropping soils both in summer and winter.

Changes in the composition of fungal communities in long-term continuous cropping are mainly driven by root exudates (Yim et al., 2020; Rose, Parker & Punja, 2003; Botnen et al., 2020). An increasing number of studies have also demonstrated that unbalanced soil nutrition, deterioration of physical and chemical properties of soil, accumulation of autotoxic substances are other important factors influencing fungal communities (Bui et al., 2020; Frac et al., 2020; Morales Moreira, Helgason & Germida, 2020). In the present study, alpha diversity and taxonomic summary of the soil fungal community under continuous and rotational cropping practices showed that both species richness and species diversity were not affected in the winter, suggesting that the effect of rotational cropping practices on soil fungal communities was affected by season (Fig. 2). This was also confirmed by PCoA1 results (Fig. 3). The difference in fungal diversity in the summer and winter may be attributable to the different nutrient and water requirements of pineapple during growth and development in different seasons, which affect the types and quantities of root exudates. Moreover, RDA analyses of winter samples showed that rotational cropping reduced the correlation between TN and available P and the fungal community (Fig. 5). These results provide a solid foundation for the choice of rotational cropping practices in the summer to elevate fungal diversity, which will contribute to the yield of pineapple.

The LEfSe algorithm is a tool for the discovery of metagenomic microbial biomarkers by analyzing the abundance of microbes (Pedamallu et al., 2016). In the present study, several fungal biomarkers were identified, including *Sporobolomyces* and *Aspergillus* at the genus level, *Penicillium* and fungal sp. p1s11 at the species level in rotational cropping soil at Xuwen in winter; Corynascus sp. JHG 2007 and Corynascus at the genus level in rotational cropping soil in Xuwen in summer; ales family Incertae sedis in continuous cropping soil at Xuwen in winter; and Sordariomycetes at the class level in continuous cropping soil at Xuwen in summer (Fig. 6). The relative abundance of these biomarkers changed significantly after rotational cropping in Xuwen in both summer and winter seasons. It has been demonstrated that Sporobolomyces is the predominant species in several kinds of soil samples (Slavikova & Vadkertiova, 2003; Li et al., 2020). Phosphate solubilization of *Penicillium oxalicum* P4 and *Aspergillus niger* P85 in calcareous soil can promote maize growth (Yin et al., 2015). The diversity of Aspergillus and Penicillium in the soil of the Brazilian tropical dry forest could contribute to environmental preservation (Barbosa et al., 2016). PevD1 from Sordariomycetes can target cotton PR5-like proteins and promote fungal infections (Zhang et al., 2019). The results of this study demonstrate that fungi in rotational cropping soil may contribute to increased pineapple production by enhancing plant growth and promoting environmental diversity in the planting soil.

We also noticed that different rotation systems might have different impact on soil fungal communities. In Leizhou, approximate 60% farmlands used pineapple–banana rotation cropping, and the left used pineapple–capsicum cropping. While in Xuwen, almost all pineapple rotational farmlands used pineapple–sugarcane cropping. Although no significant difference was observed for the physical and chemical characteristics of soil in different rotational fields (Chen, Gong & Xu, 2020), the rotational farms at Leizhou demonstrated significantly higher alpha diversity indices than those at Xuwen in summer (Fig. 2). The different rotation systems between Xuwen and Leizhou also led to different abundance in some specific fungal taxa. We found six fungal biomarkers in rotational cropping soil at Xuwen. In contrast, no fungal biomarkers were found in Leizhou in rotational cropping soil both in summer and winter. Biomarkers of rotational cropping soil at Xuwen in winter (*Sporobolomyces*, *Aspergillus*, *Penicillium*, and *fungal sp. p1s11*) were highly abundant in the corresponding groups at Leizhou, whereas the biomarkers of XRS group (*Corynascus sp. JHG 2007* and *Corynascus*) were less abundant in LRS group. We inferred that although no obvious difference was observed in the alpha diversity indices between pineapple–banana and pineapple–capsicum rotation cropping farms at Leizhou (Table S3), the two different rotation systems might lead to the differential distribution of some specific fungal taxa at this location. This inference will be investigated further in subsequent studies.

## Conclusion

In summary, both species richness and species diversity were affected by rotational cropping in summer, but not in winter. *Ascomycota, Basidiomycota, Zygomcota*, *Chytridiomycota*, *Chaetomium, Penicillium, Fusarium, Trichoderma*, and *Cryptococcus* were the dominant fungal phyla and genera in the continuous and rotational cropping soil of pineapple both in summer and winter. *Chytridiomycota* at the phylum level and *Gibberella* at the genus level were observed after rotational cropping in both winter and summer. However, *Ascomycota* at the phylum level and *Chaetomium* at the genus level were the most abundant fungal species in continuous cropping soil both in Xuwen and Leizou in summer. Several fungal biomarkers were also identified in Xuwen, both in the continuous and rotational cropping soil in summer and winter. These results provide a foundation for understanding the effects of fungi that are associated with continuous cropping, application of microbial fertilizers, and/or soil amendments on pineapple plant. This understanding can help in the development of strategies against problems associated with continuous cropping.

## Supplemental Information

10.7717/peerj.13937/supp-1 Supplemental Information 1 Supplemental tables.

## Additional Information and Declarations

### Field Study Permissions

The following information was supplied relating to field study approvals (*i.e*., approving body and any reference numbers): Field experiments were approved by the Chinese Academy of Tropical Agricultural Sciences (Approval No. 17CXTD-10).

### Data Availability

The following information was supplied regarding data availability: The ITS amplicon sequencing reads are available in the Sequence Read Archive (SRA) BioProject: PRJNA786445. https://www.ncbi.nlm.nih.gov/bioproject/PRJNA786445.
